# The ionospheric irregularities climatology over Svalbard from solar cycle 23

**DOI:** 10.1038/s41598-019-44829-5

**Published:** 2019-06-25

**Authors:** Giorgiana De Franceschi, Luca Spogli, Lucilla Alfonsi, Vincenzo Romano, Claudio Cesaroni, Ingrid Hunstad

**Affiliations:** 1 Istituto Nazionale di Geofisica e Vulcanologia, Via di Vigna Murata 605, 00143 Rome, Italy; 2 SpacEarth Technology Srl, Via di Vigna Murata 605, 00143 Rome, Italy

**Keywords:** Aurora, Magnetospheric physics

## Abstract

The paper presents an unprecedented description of the climatology of ionospheric irregularities over the Arctic derived from the longest Global Navigation Satellite Systems data series ever collected for this specific aim. Two TEC and scintillation receivers are working at Ny-Ålesund (Svalbard, NO), the first of which has been installed in late September 2003. They sample the L1 and L2 signals at 50 Hz from all the GPS satellites in view. The receivers monitor an area of about 600 km radius that includes the auroral and cusp/cap regions in the European longitudinal sector. The length of the data series and the privileged site of observation allow describing the Arctic ionosphere along about two solar cycles, from the descending phase of cycle 23 to almost the end of cycle 24. Our analysis results into a detailed assessment of the long-term behaviour of the ionosphere under solar maximum and solar minimum conditions, including several periods of perturbed ionospheric weather caused by unfavourable helio-geophysical conditions. Since November 2015, a multi-constellation GNSS receiver has been deployed in Ny-Ålesund, providing the opportunity to perform the ionospheric climatology from Galileo signals. The results offer realistic features of the high latitude ionosphere that can substantially contribute to the necessary improvements of forecasting models, providing a broad spectrum of ionospheric reactions to different space weather conditions.

## Introduction

The interest about the impact of the ionosphere on the propagation of Global Navigation Satellite Systems (GNSS) signals is continuously increasing mainly because of the high demand of precise positioning (with positioning error under one meter). The ionosphere induces two effects on the received signals: a delay due to the presence of free electrons, and diffraction and refraction caused by the uneven distribution of the electron density and by the plasma velocity changes. The latter effect causes fluctuations on the phase and the amplitude of the received signals. The fluctuations due to diffractive effects are termed scintillations. The scintillation occurrence is highly variable in space and time and depends on local time, season, solar and magnetic activity. Scintillations are frequent at low latitudes, where, generally, they occur daily during the post-sunset hours, and at high latitudes where they can appear at any time, often associated with the geomagnetic storms^1–3^, but happening also at quiet times^4,5^. Differently from phase scintillation, the amplitude scintillation is biased by irregularities probing size (on L band) of hundreds meters^6,7^. The first literature on the scintillation climatology at high latitudes reported a prevalence of phase scintillations^4^. Such results derived from the data analysis made applying a filter with a fixed cut-off frequency at 0.1 Hz to compute σ_Φ_, the phase scintillation index. The σ_Φ_ is derived from the standard deviation of the detrended carrier phase^8^. After the papers by Forte^9,10^, Forte and Radicella^11^ and Beach^12^, some more recent papers^13,14^ report that this choice of cut-off frequency is not always able to remove the refractive (deterministic) effects at high latitude. This issue opened a debate: do we have to revise the use of the term scintillation in the existing literature? Considering the scintillation a stochastic process we have to call scintillations only those amplitude and phase fluctuations due to diffractive (stochastic) effects (not removable from the data). The idea to investigate only the scintillations is surely acceptable in a space weather context, in which the scintillation is seen as an effect of degradation of the received signal. In this frame, in fact, the scope is to nowcast, forecast and, possibly, mitigate the scintillations. Nevertheless, the determination of the optimal choice of the cut-off frequency to be adopted to describe the scintillation climatology is challenging. In the next section, we demonstrate how difficult is to choose the “best” cut-off frequency to remove the refractive effects on a long-term data set. On the other hand, if the scope of the analysis is to retrieve information on the dynamics and fragmentation of the ionospheric plasma causing phase and amplitude fluctuations on the received signals, we think that it is worth keeping the refractive effects in the climatology description. In this framework, hereafter we refer to scintillations when the events of high σ_Φ_ are accompanied by high amplitude scintillation index, S4. S4 is the standard deviation of the received power normalized by its mean value. When the enhancement is recorded only on the occurrence of σ_Φ_ above a fixed threshold, we refer to refractive effects identifying changes in the ionospheric plasma velocity and presence of irregularities with scale sizes larger than the first Fresnel’s radius. We consider this information crucial to understand the physics ruling the extremely dynamical and complex environment resulting from the plasma response to the electric fields of magnetospheric origin^15^. At high latitudes, the formation and the dynamics of the ionospheric irregularities is strictly linked to the configuration assumed by the convection pattern of the ionosphere as a result of the interplay between the geomagnetic field and the Interplanetary Magnetic Field (IMF)^15,16^. The solar activity further affects this interplay by modulating the compression of the solar wind (in which the IMF is considered frozen in) on the dayside Earth’s magnetic field. The mapping of the Earth’s magnetic field lines describes the auroral oval^17^ that, under stormy conditions, shows a different configuration from the one assumed in quiet times. The whole phenomenology is translated in a fast moving plasma (with typical velocities up to 1000 m/s and more), characterized by acceleration, deceleration and stagnation^18^ and by highly variable electron density structuring. Such conditions can, indeed, give rise to refractive effects on GNSS signals recorded at auroral and polar latitudes.

In the last decade, several authors investigated the high latitude L-band scintillation under severe stormy conditions, thanks to the increased capability to monitor the high latitude ionosphere offered by ground based GNSS receivers, also combined with optical and radio experimental observations. These investigations discussed the temporal and spatial occurrence of the typical enhanced scintillations due to the most effective storms in the solar cycles 23 (e.g. the Halloween storms^18^) and 24 (e.g. the Saint Patrick’s day storm^19^). The main outcome refers to the tongue of ionization, and to storm enhanced density plume as the cause of enhanced phase scintillation. Climatological studies on L band scintillations at high latitude started to appear later on^5,20–23^ limited to few years of data availability. The high degree of unpredictability of the polar and auroral scintillations by global models^24^ makes a detailed climatological description of the ionosphere at L-band over at least one solar cycle very challenging. In turn, the availability of continuous monitoring of the high latitude scintillations provides information that can be used as input, e.g., to improve the global models’ performance, particularly under severe space weather conditions^25^.

Bearing this in mind, we use the longest series of data from two GSV4004 GPS Ionospheric Scintillation Total Electron Monitors (GISTMs^8^) ever collected. The first unit was deployed at Ny-Ålesund, Dirigibile Italia Station (78.923°N, 11.925°E), in September 2003^26^. A second GISTM was installed in November 2006 in the Statens Kartverk (78.929°N, 11.864°E), about 1 km apart. Here we present a climatology of ionospheric irregularities and scintillations based on the data collected from September 2003 to December 2016 by both receivers. The time interval includes the descending phase of the 23^rd^ solar cycle and the 24^th^ solar cycle. The data availability during the considered period is good, although a few gaps between 2009 and 2010 (during the solar minimum) and in 2013 (during the solar maximum). Taking the opportunity offered by a PolaRxS receiver deployed in Ny-Ålesund, Dirigibile Italia station, in November 2015, we compare the irregularities and the scintillation scenario as derived from GPS and Galileo data acquired during the entire 2016. We refer to the same interval to investigate the impact of the cut off frequency choice on the determination of scintillation occurrence.

**Figure 1:**
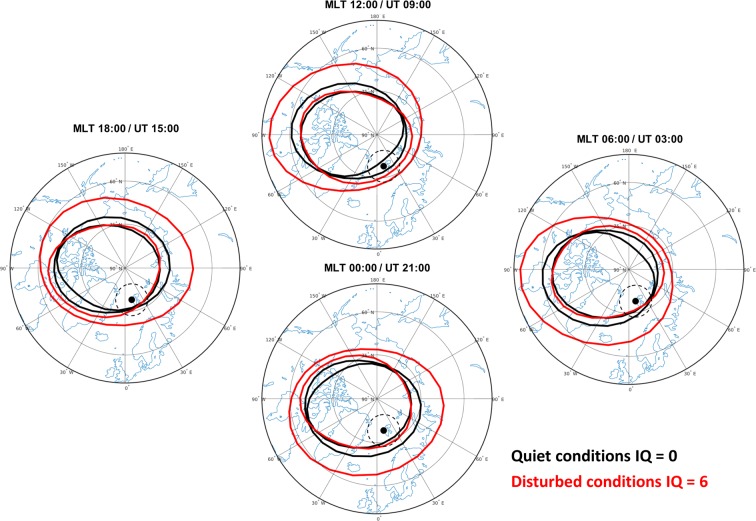
Position of the poleward and equatorward boundaries of the Feldestein auroral oval for quiet (IQ = 0, black lines) and disturbed conditions (IQ = 6, red lines) at 12 MLT (top), 6 MLT (right), 00 MLT (bottom) and 18 MLT (left). The corresponding UT time is also reported in the figure. Dashed lines indicate the field of view at 350 km spanned by the receivers located in Ny-Ålesund (black dot) considering an elevation angle mask of 30°.

As from Fig. 1, the field of view of the two receivers includes the auroral and polar cap regions, identified by a climatological description of the poleward and equatorward boundaries of the Feldestein auroral oval^27,28^ for quiet (IQ = 0, black lines) and disturbed conditions (IQ = 6, red lines). The observing site, in fact, includes the regions inside the ovals and inside the cap, depending on the Magnetic Local Time (MLT) and the disturbance level^4,27^. The difference between MLT and Universal Time at Svalbard is 3 hours, as indicated also in the figure, while the geometry of the modelled auroral oval depends on the coupling among solar wind, magnetosphere and high-latitude ionosphere. This peculiar position of the observing site allows looking at ionospheric irregularities caused by particle precipitation into the ionosphere and by polar cap patches^18^. The next section is dedicated to the data and the adopted method of analysis. The last section deals with the presentation and discussion of the results achieved so far.

## Data and Methods

### Scintillation and TEC data

The GSV4004 GISTM^8^ consists of a NovAtel OEM4 dual‐frequency receiver with a special firmware that provides in near real time, from 50 Hz sampling, the amplitude and the phase scintillation from the GPS L1 (1575.42 MHz) frequency signal, and the ionospheric Total Electron Content (TEC) from the GPS L1 and L2 (1227.6 MHz) carrier phase signals. The device includes a firmware that provides the amplitude and phase scintillation indices, S4 and σ_Φ_. In our analysis, we consider the scintillation indices computed over 60 s. The occurrence of ionospheric scintillation is associated with the presence of plasma irregularities, which are regions where the electrons concentration is significantly different from the surrounding areas. The TEC gradients reveal the presence of ionospheric irregularities and their spatial and temporal variation highlights the plasma dynamics^18^. Thus, besides the analysis of the long-term variation of the scintillation indices, we consider also the climatology of the Rate Of TEC change (ROT).

The receiver firmware provides the indices obtained by: (1) a high‐pass filter for the detrending of the raw power and phase measurements, and (2) a Butterworth filter with a default choice of a constant cut-off frequency at 0.1 Hz^11,12,28^. Recent studies^28^ demonstrate that the large variety of plasma drift velocity occurring at high-latitudes may require adaptive values of the cut-off frequency to disentangle diffractive (stochastic) phase variations from refractive (deterministic) phase variations. The known effect of this is a reduction of the “phase-without-amplitude scintillation occurrence^28,29^”. However, a standard approach has not yet been identified and an optimum detrending has not yet been implemented in the firmware of commercial receivers for scintillation monitoring. To date, different choices of cut-off frequency have been proposed on the base of some case studies^13,14,28^, hardly transferrable on long-term statistics.

In this work, we concentrate on the information that can be derived by comparing amplitude and phase scintillation indices. In fact, the amplitude and phase response of an electromagnetic wave to structure is determined by Fresnel filtering, which strongly suppresses the amplitude contribution for scales larger than the Fresnel scale^4^. Moreover, σ_Φ_ is very sensitive to plasma dynamics and it is useful in estimating the signal fluctuations caused by ionospheric irregularities larger than the Fresnel scale, which may even lead to signal loss of lock^9^. Thus, when comparing the different behavior of S4 and σ_Φ_ occurrences it is possible to speculate about the scales of the irregularities involved in the resulted scintillation patterns and on the different velocity regimes assumed by the plasma.

The PolaRxS receiver extends the capability of the GSV4004 providing, among the other features, the multi-constellation tracking ability. In fact, PolaRxS is able to track simultaneously GPS L1CA, L1P, L2C, L2P, L5; GLONASS L1CA, L2CA; Galileo E1, E5a, E5b, E5AltBoc; COMPASS B1, B2; SBAS L1^30^. It provides the same quantities given by the GSV4004, but for all the frequencies and constellations, and the measure of the spectral parameters: spectral slope of the phase Power Spectral Density (*p*) in the 0.1 to 25 Hz range and the spectral strength of the phase PSD (*T*) at 1 Hz (60 s).

### The Ground Based Scintillation Climatology

The climatology of the expected features of ROT, of its standard deviation (ROT SD) and of the percentage occurrence of σ_Φ_ and of S4 as a function of MLT and MLAT is obtained by applying the well-established GBSC (Ground Based Scintillation Climatology) technique^4,23,31,32^. GBSC takes in input the measurements of different kind of GNSS receivers, by ingesting scintillation as well as TEC/ROT data. In this work, the considered outputs of the GBSC are maps in MLT and Altitude Adjusted Corrected Geomagnetic^33^ Latitude (AACGMLat) (bin size: 1° × 1 hour) of the following quantities:

- Percentage occurrence of S4 and σ_Φ_ above the weak (0.1), moderate (0.25) and strong (0.7) regime thresholds (units are radians for σ_Φ_). The percentage occurrence *O* is evaluated in each bin (*i*_*MLT*_,*j*_*MLAT*_) as $O({i}_{MLT},{j}_{MLAT})=\frac{{N}_{thr}({i}_{MLT},{j}_{MLAT})}{{N}_{tot}({i}_{MLT},{j}_{MLAT})}$, in which *N*_*thr*_*(i*_*MLT*_*,j*_*MLAT*_) is the total number of the considered scintillation indices above the considered threshold and *N*_*tot*_*(i*_*MLT*_*,j*_*MLAT*_) is the total number of observation available in the bin;
- Mean and standard deviation of ROT in each bin (*i*_*MLT*_,*j*_*MLAT*_).

To give a representation of the scintillation conditions as independent as possible of the relative geometry between the satellites and the receiver, the scintillation indices have been both projected to the vertical (σ_Φ_^*vert*^, S4^*vert*^) according to the following formulae^4,23,31,32^:

1 $${\sigma }_{{\rm{\Phi }}}^{vert}=\frac{{\sigma }_{{\rm{\Phi }}}^{slant}}{F{({\alpha }_{elev})}^{0.5}}$$

2 $$S{4}^{vert}=\frac{S{4}^{slant}}{F{({\alpha }_{elev})}^{\frac{p+1}{4}}}$$

in which σ_Φ_^*slant*^ and S4^*slant*^ are the 1-minute indices as measured by the receiver along the slant path. Hereafter, the values of the scintillation indices (σ_Φ_, S4) refer to their vertical values. The verticalization process does not apply to ROT. In formulae (1) and (2), *p* is the spectral slope of the phase Power Spectral Density and *F(α*_*elev*_) is the obliquity factor^34^, which is function of the elevation angle *α*_*elev*_ at which the observation is made, of the Earth’s radius (*R*_*E*_) and of the altitude of the Ionospheric Pierce Point (*H*_*IPP*_):

3 $$F({\alpha }_{elev})=\frac{1}{\sqrt{1-{(\frac{{R}_{E}\cos {\alpha }_{elev}}{{R}_{E}+{H}_{IPP}})}^{2}}}$$

Two assumptions are implicit in the verticalization^32^: (i) the ionosphere is treated as a single layer (assumed to be located at *H*_*IPP*_ = 350 km), which allows to write the exponent of *F(α*_*elev*_) in Eq. (2) as $\frac{p+1}{4}$ and (ii) the scintillation regime is weak, which allows to write the verticalization in the form of Eqs (1) and (2)^35^. All the *caveat* in applying such approximation of statistical studies about scintillation are critically discussed in section 3 of the work by Spogli and co-authors (2013)^32^. Here we remind that such approximation may lead to an underestimation of the scintillation occurrence, affecting mainly the values close to the threshold between weak and moderate scintillation. The assumptions may not hold during extreme events, nevertheless for climatology purposes it suffices. The assumption of locating the *H*_*IPP*_ at 350 km supposes that the bulk of the ionospheric variations come from the F region. Unfortunately, in the considered area there are not ancillary instruments (e.g. ionosonde) useful to resolve the altitude on long-term basis, hence supporting the height discrimination. At climatology level we do not expect a significant effect of such a choice on our mapping.

While the exponent $\frac{p+1}{4}$ can be directly estimated by the *p* measurements provided by PolaRxS, for GSV4004 a fixed value of *p* = 2.6 has been adopted^22^. To remove the effect of the multipath due to the environment surrounding the receiver, possibly leading to mis-identification of the scintillation events, an elevation angle mask of 30° is applied on the data.

The considered GSV4004 data runs from 1 October 2003 to 31 September 2016 (with some data gaps in 2009 and 2013), while the PolaRxS data refers to the whole year 2016, without meaningful data gaps. However, we do not expect significant impact of such gaps in a very long data series.

### IMF and Kp conditions

**Figure 2:**
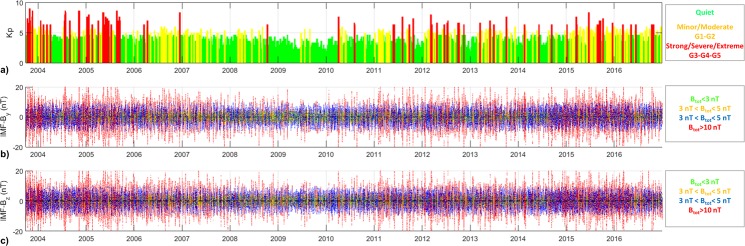
Time profile of Kp (**a**), IMF-By (**b**) and IMF-Bz (**c**). Different colors indicate different ranges of each values according to the legend reported in the right part of each plot.

**Figure 3:**
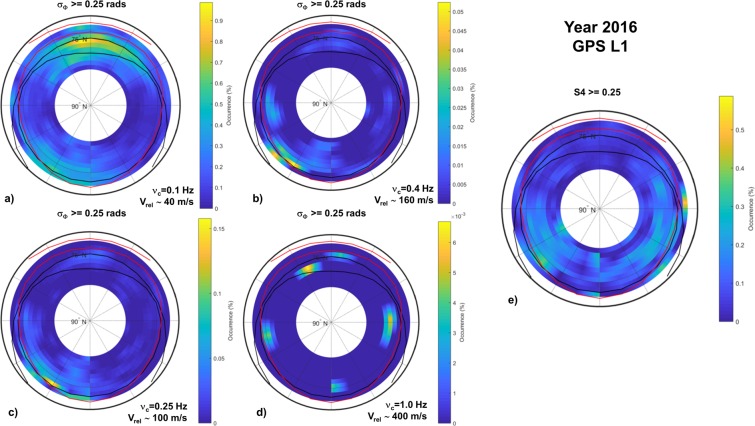
Climatology maps of occurrence of σ_Φ_ ≥ 0.25 radians obtained by using a cutoff frequency ν_c_ of 0.1 Hz (panel a), 0.25 Hz (panel c), 0.4 Hz (panel b) and 1.0 Hz (panel d). (Panel e) Climatology map of occurrence of S4 ≥ 0.25. All maps refers to GPS observations on 2016.

The maps obtained by means of the GBSC technique are sorted according to the geomagnetic storm intensity (according to the Kp index) and according to the solar wind conditions (according to the IMF B_*z*_ and B_*y*_ components). The Kp, expressed here in terms of NOAA G scale, allows identifying the dependence on the geomagnetic field disturbance level in the sub-auroral regions of the globe due to the solar particle radiation^35,36^ (www.swpc.noaa.gov/noaa-scales-explanation). The G scale ranges from 1 to 5, i.e. from minor to extreme storm, identified by Kp = 5 and Kp = 9, respectively. Figure 2a reports the time profile of the Kp index (3-hourly value) along the considered period, highlighting in green, yellow and red the periods in which the conditions were quiet (G0), minor/moderate (G1/G2) and strong/severe/extreme (G3/G4/G5), respectively. As expected, the most disturbed conditions occurred during the maximum and the ascending/descending phases of the solar cycle. Figure 2b,c report the time profile of the IMF conditions along the considered period, characterized on the base of the measurements provided by the Advanced Composition Explorer (ACE) spacecraft, orbiting around the Lagrangian L1 point. The data are part of the OMNI_HRO_1MIN dataset provided by Coordinated Data Analysis Web site and include 1-minute values of the IMF components plus total field shifted to the nose of the Earth’s bow shock. Panels b and c of Fig. 3 report the time profile of IMF-By and IMF-Bz, respectively. The colours indicate different ranges of the total field Btot, inspired to those suggested by the Super Dual Auroral Radar Network (SuperDARN) community^37^.

Kp data are available from the website of the World Data Center for Geoagnetism, Kyoto (http://wdc.kugi.kyoto-u.ac.jp). IMF data are available at Coordinated Data Analysis Web site (cdaweb.sci.gsfc.nasa.gov). All the scintillation and ROT data are downloadable from the eSWua website (electronic Space Weather upper atmosphere, www.eswua.ingv.it).

## Results

### Study of the cut-off frequency for phase detrending

To look for the “optimal” phase detrending on long-term data series we have compared the occurrence of σ_Φ_, computed adopting different cut-off frequencies, with the occurrence of S4, by analysing the GPS raw data provided by the PolaRxS along the entire 2016. The results are described in the GBSC maps given in Fig. 3. The figure is composed by climatology maps of occurrence of σ_Φ_ ≥ 0.25 radians obtained by using different choices of the cut-off frequency ν_c_ to filter the Power Spectral Density of the phase spectrum, namely 0.1 Hz (panel a), 0.25 Hz (panel c), 0.4 Hz (panel b) and 1.0 Hz (panel d). Over these maps, and over all other maps in this paper, we superimpose the auroral oval boundaries given by the Feldstein, Holzworth and Meng model^35,36^ for quiet and disturbed magnetic activity levels (IQ = 0 and IQ = 6, respectively), to indicate the approximate position of the oval and its modelled displacement due to geospace forcing.

The comparison is made with the climatology map of occurrence of S4 ≥ 0.25 (Panel e) to look for the moderate to strong scintillation regimes. The effect of choosing a greater cut-off frequency results in a significant decrease of the phase scintillation occurrence (visible in the different occurrence scales). Comparing the σ_Φ_ occurrence at different cut-off frequencies with the S4 occurrence, it seems that the choice of ν_c_ at 0.25 and at 0.4 Hz maximize the similarity with the S4 occurrence map. The regions highlighted both in the σ_Φ_ and the S4 maps identify the events of phase scintillation accompanied by amplitude scintillation, mainly concentrated in the MLT afternoon/evening sector. Nevertheless, the S4 occurrence and the σ_Φ_ occurrence are still substantially different, regardless the ν_c_ choice. The exercise shows well how difficult is to choose the “best” cut-off frequency to remove the phase without amplitude scintillation events at a climatological level. Ideally, this would be possible only having a simultaneous information of the plasma relative velocity and on the irregularities scale sizes through actual measurements from ground-based stations (e.g. SuperDARN) and/or from *in-situ* satellites (e.g. Swarm). Unfortunately, this is not possible because of the scarce temporal and spatial coverage of the desired data, on the limitation of the devices (e.g., HF absorption during storms affecting SuperDARN radars) and the peculiar geometry of the observation needed. So the most promising way to provide a detailed long-term description up to now of the ionospheric irregularities over Ny-Ålesund is to keep a fixed cut-off frequency, bearing in mind the perils of such a choice. For the remainder of the paper, we deliberately adopt a fixed cut-off frequency at 0.1 Hz. Hereafter, we will refer to scintillations when they appear simultaneously on the phase and on the amplitude of the GNSS signals and we refer to electron density fluctuations due to refractive effects when they appear only on the phase of the received signals. Such approach allows describing the physics ruling the ionospheric irregularities origin, formation and dynamics at Svalbard from solar cycle 23 to 24.

### Overall irregularities climatology

**Figure 4:**
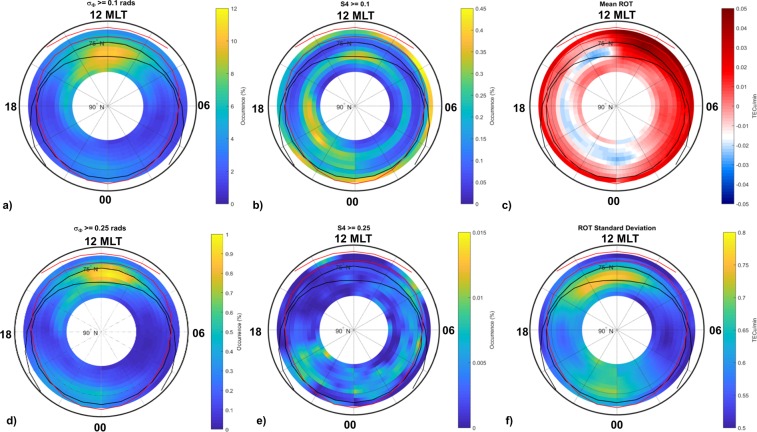
Scintillation and ROT climatology maps obtained from the full dataset: maps of occurrence of σ_Φ_ above 0.1 (**a**) and 0.25 (**d**) radians; maps of S4 above 0.1 (**b**) and 0.25 (**e**) maps of mean (**c**) and standard deviation of ROT (**f**).

The ionospheric climatology derived from the entire dataset collected at Ny-Ålesund is presented in form of polar maps, MLT vs. AACGMLat. Independently on the helio-geophysical conditions, Fig. 4 gives a full panorama of the resulting percentage occurrence of σ_Φ_ (panels a,d) and S4 (panels b,e) above two selected thresholds: 0.1 (given in rad for σ_Φ_ and unit-less for S4) and 0.25 (given in rad for σ_Φ_ and unit-less for S4), to account for weak-to-strong and moderate-to-strong scattering condition, respectively. Panels c and f report the maps of the mean ROT and its standard deviation (ROT SD), respectively.

As expected, the percentage occurrence of S4 is significantly lower than that one of σ_Φ_, from weak to strong scattering regime (Fig. 4 panels a,b and b,c). Their maxima (yellow in the colour bar) are differently distributed in space: S4 shows a scattered spatial distribution while σ_Φ_ results to bulk clearly in the cusp region around the magnetic noon. Such discrepancy is surely linked to the choice of keeping a fixed cut-off frequency at 0.1 Hz, but allows to identify the scintillations in the regions where σ_Φ_, and S4 occurrences increase simultaneously and ionospheric conditions producing refractive effects where only the σ_Φ_ occurrence enhances. The latter testifies the setup of physical conditions in which the ionosphere is highly dynamic, characterized by large scale size irregularities moving at different velocities with respect to the ambient ionosphere.

The climatology of the weak to moderate scintillations (σ_Φ_ ≥ 0.1 rad and S4 ≥ 0.1) evidences the largest occurrence in the quiet (IQ = 0) auroral oval and at the cusp around noon (Fig. 4a,b). In the same MLT sector, the refractive effects are found at higher latitudes, within the polar cap (within the black inner boundary). They are still present even when the σ_Φ_ minimum threshold is at 0.25 rad (Fig. 4d). The most severe scintillations (σ_Φ_ ≥ 0.25 rad and S4 ≥ 0.25) are mainly confined in the pre-midnight polar cap (Fig. 4d,e). While significant refractive effects (σ_Φ_ ≥ 0.25 rad) are visible at noon.

On the average, the negative TEC gradients (mean ROT, Fig. 4c) occur mainly in the afternoon/evening sector: from noon, within the quiet auroral oval, to midnight, in the cap. Whilst the positive TEC gradients result to characterize principally the auroral and the sub-auroral regions in the morning and the sub-auroral zones during night-time. As expected, the ROT standard deviation correlates with the scintillation occurrence and with the refractive effects on the signals: it maximizes within the oval around noon-early afternoon, and in the pre-midnight sector at all latitudes (Fig. 4f). The ROT standard deviation at noon well correlates with the refractive effects identified by σ_Φ_ and are in agreement with what found by^38^.

To further detail the distribution of TEC gradients and of scintillations occurrence, we applied the climatology to sub-data sets, sorted according to the solar cycle, global geomagnetic storm index Kp and to the different conditions of IMF.

### Irregularities climatology dependence on solar cycle

**Figure 5:**
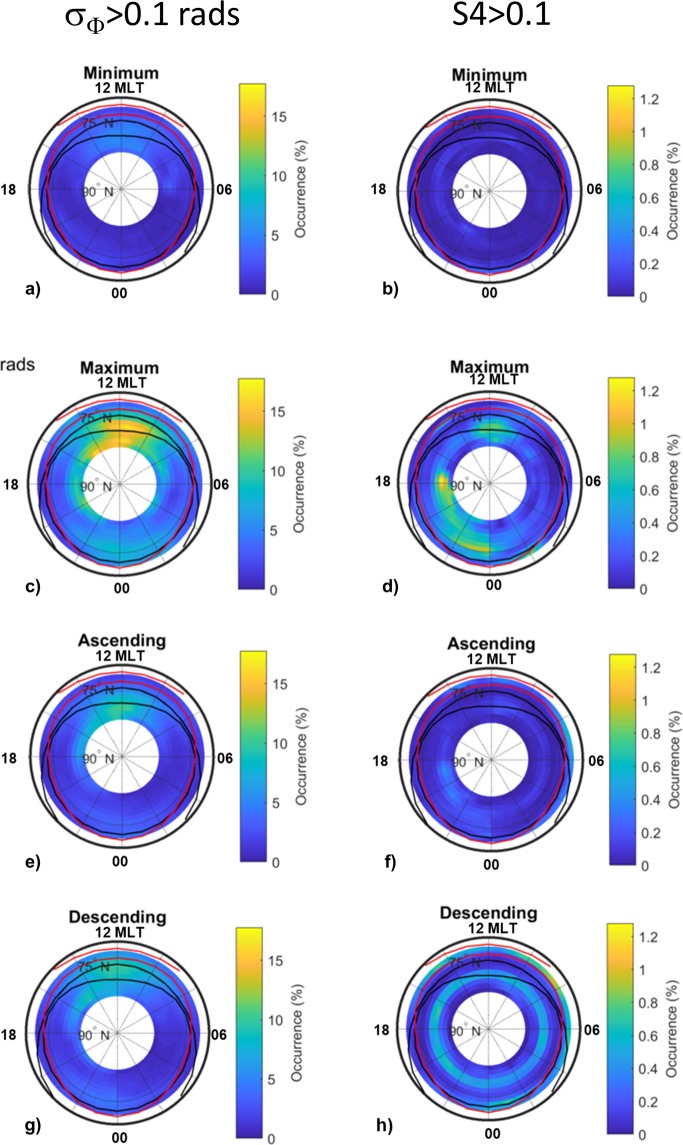
Scintillation and irregularities climatology maps obtained from the full dataset sorted according minimum, maximum, ascending and descending phase of the solar cycle: maps of occurrence of σ_Φ_ above 0.1 (**a**–**d**) radians; maps of S4 above 0.1 (**e**–**h**).

To learn more about the dependence of scintillations and of irregularities producing refractive effects on the solar activity, we have sorted the data according to the minimum (2007–2009), ascending (2010–2012), maximum (2013–2015) and descending (2003–2006) phases of the solar cycle. The results given in Fig. 5 confirm that during the years of solar minimum the scintillations occur rarely and located around the inner boundary of the quiet auroral oval or within the cap from noon to afternoon/evening MLT hours. In the same phase of the cycle, the ionospheric conditions producing refraction populate largely the cusp. In the ascending phase, the pre-noon and the evening sectors are the regions hosting the scintillations, while the refractive effects appear from (quiet) auroral latitudes to the cap around noon and well within the cap during the MLT afternoon. When the solar activity reaches its maximum, the scintillations appear at auroral latitudes around noon and in the polar cap from the afternoon to the post-midnight sector. During the descending phase of the solar cycle, the occurrence of scintillation maximizes around noon and midnight at the (quiet) auroral oval boundaries. Refractive effects are visible during the afternoon in the cap.

### Irregularities climatology dependence on geomagnetic storm intensity

For our purpose, we sorted the ionospheric data into three classes: quiet conditions (Kp < 5), minor/moderate conditions (5 ≤ Kp ≤ 6), strong/severe/extreme conditions (Kp > 6).

**Figure 6:**
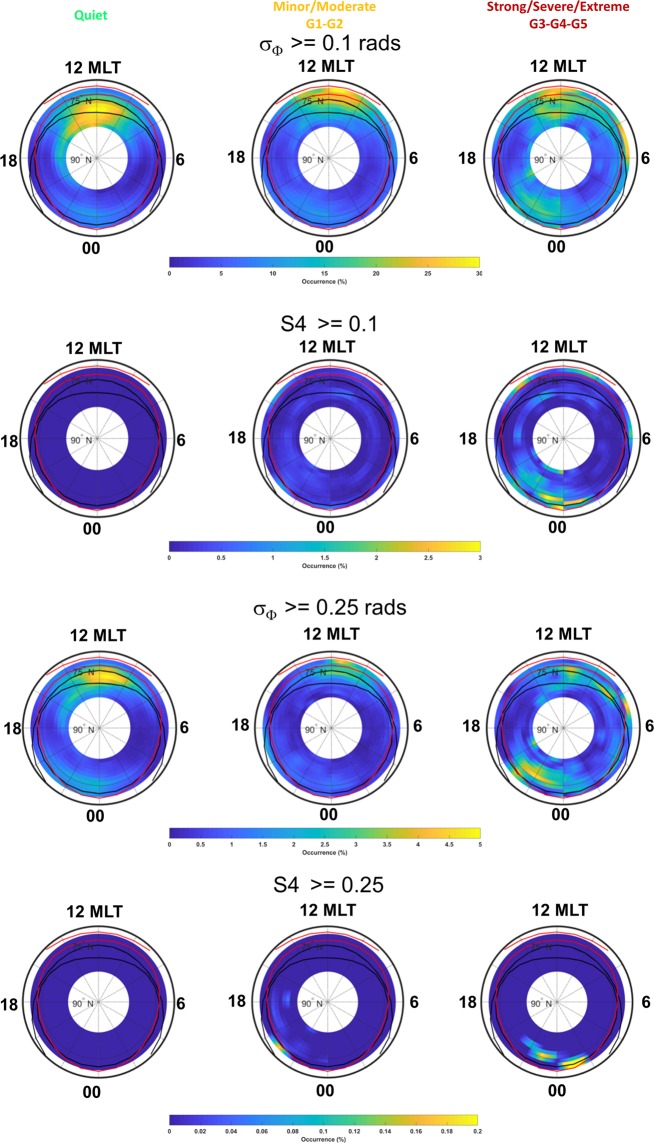
Scintillation (from weak to strong, top plots, and from moderate to strong, bottom plots, scintillation occurrence of σ_Φ_ and S4) and ROT climatology maps sorted according different level of Kp: quiet (left plots), minor/moderate G1-G2 (middle plots) and strong/severe/extreme G3-G4-G5 (right plots).

Figure 6 shows (from top to bottom) maps of: percentage occurrence of σ_Φ_ ≥ 0.1 radians, percentage occurrence of S4 ≥ 0.1, percentage occurrence of σ_Φ_ ≥ 0.25 radians, percentage occurrence of S4 ≥ 0.25, separately for the three classes of geomagnetic storm intensity conditions (quiet, minor/moderate, strong/severe/extreme).

The distribution in MLT of the σ_Φ_ occurrence appears dependent on the level of geomagnetic activity especially in the midnight sector. Under all the geomagnetic conditions, the hours most affected by scintillations are in the evening/midnight sector. The refractive effects are mainly visible around noon, regardless the intensity of the storms. With increasing geomagnetic storm intensity, these effects shift from the polar and quiet auroral region towards the lower latitudes, mainly confined in the disturbed auroral oval (G1, G2) with signatures at higher latitudes in the polar cap under strong-extreme conditions (G3, G4, G5). With increasing G, it is worth noticing the enhancement of scintillations in the evening/pre-midnight sector at all latitudes. This is likely due to the nightside reconnection. When the indices threshold increase (σ_Φ_ ≥ 0.25 rad and S4 ≥ 0.25) the severe scintillations appear in the midnight hours (with a preference for the pre-midnight) at the inner boundary of the auroral oval, especially under G3-G5 conditions.

### Irregularities climatology dependence on solar wind conditions

The high latitude ionosphere is strongly influenced by the solar wind and IMF conditions that play a role on the occurrence of plasma irregularities and related scintillation phenomena facilitating, under certain conditions, the particles precipitation into the ionosphere and driving the convection pattern configuration. The IMF components B_z_, B_y_, and the total strength of the field B_*tot*_ are used for the data sorting, here inspired to that one suggested by the Super Dual Auroral Radar Network (SuperDARN) community^37^. Four intervals of *B*_*tot*_ are considered: *B*_*tot*_ < 3 nT, 3 < B_*tot*_ < 5 nT, 5 < B_*tot*_ < 10 nT and B_*tot*_ > 10 nT. For each B_*tot*_ interval is then considered the possible combination of B_*z*_ (negative/positive) and B_*y*_ (negative/positive), corresponding to four wide clock angle sectors: 45° (*B*_*z*_ > 0/*B*_*y*_ > 0), −45° (*B*_*z*_ > 0/*B*_*y*_ < 0), −135° (*B*_*z*_ < 0/*B*_*y*_ < 0) and 135° (*B*_*z*_ < 0/*B*_*y*_ > 0). The data sorting leads to four climatological maps of the percentage occurrence of σ_Φ_ >0.1 radians and of S4 > 0.1 for each *B*_*tot*_ interval.

**Figure 7:**
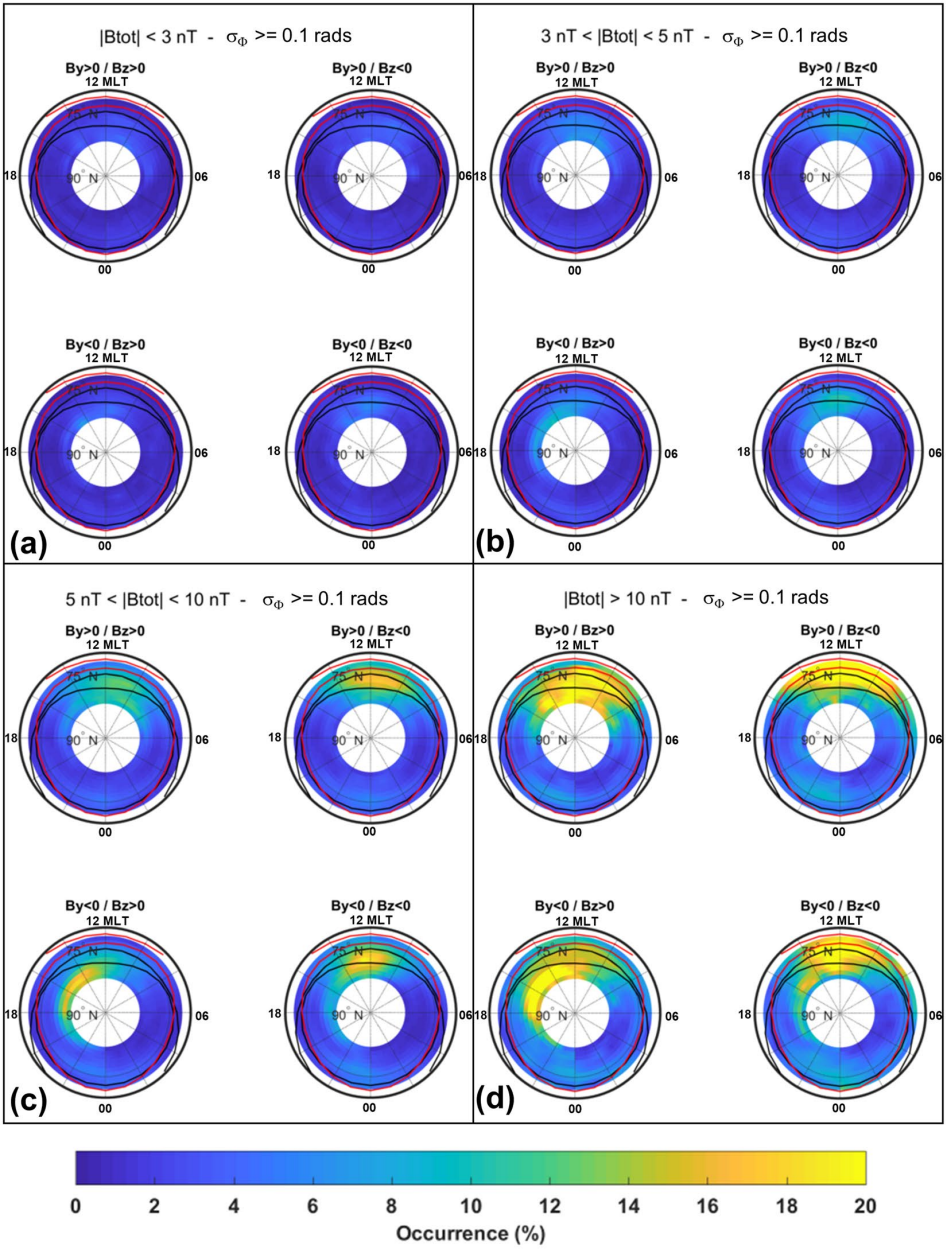
Phase Scintillation climatology under different conditions of the interplanetary magnetic field. Each panel reports the occurrence of S4 above the weak to strong scintillation threshold for each combination of By/Bz positive and negative values. Maps are sorted for different ranges of the total field |Btot|: |Btot| < 3nT (panel a), 3 nT < |Btot| 5 nT (panel b), 5 nT < |Btot| < 10 nT (panel c) and |Btot| > 10 nT (panel d).

**Figure 8:**
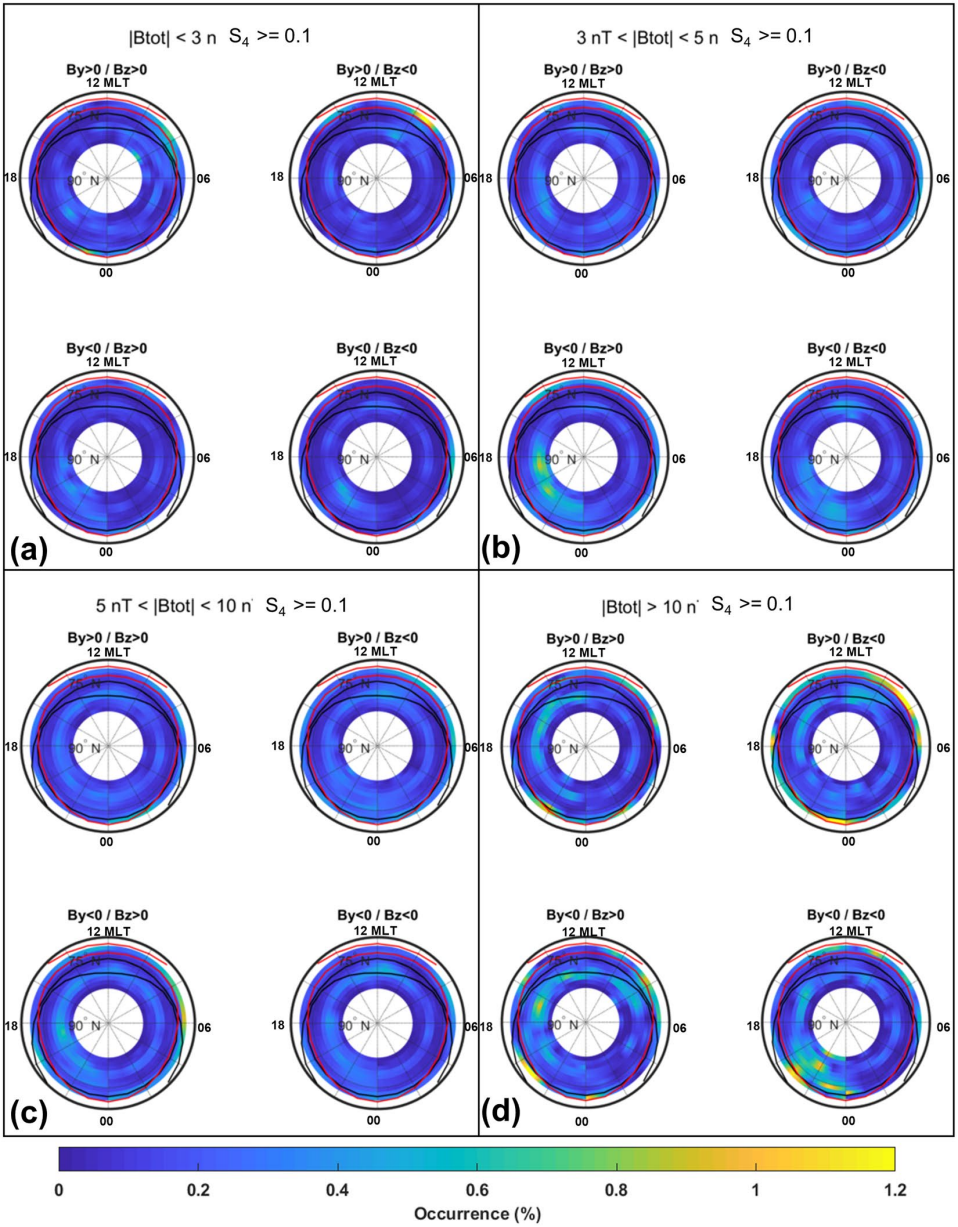
Amplitude Scintillation climatology under different conditions of the interplanetary magnetic field. Each panel reports the occurrence of S4 above the weak to strong scintillation threshold for each combination of By/Bz positive and negative values. Maps are sorted for different ranges of the total field |Btot|: |Btot| < 3nT (panel a), 3 nT < |Btot| 5 nT (panel b), 5 nT < |Btot| < 10 nT (panel c) and |Btot| > 10 nT (panel d).

Figures 7 and 8 show the resulting climatology under different B_*tot*_ magnitudes and B_*z*_/B_*y*_ conditions. Figure 7a (B_*tot*_ < 3nT), shows very low σ_Φ_ occurrence values, slowly enhancing at noon and post-noon at higher latitudes when *B*_*y*_ < 0. The occurrence raises accordingly to *B*_*tot*_: when this ranges between 3nT and 5 nT, σ_Φ_ maximizes at noon and post-noon within the cap when *B*_*y*_ is negative (Fig. 7b). Further increase of *B*_*tot*_ (5–10 nT) still highlights that the preferred time for refractive effects manifestation is around noon and post noon (when *B*_*y*_ is negative), especially in the cap region within the northward border of the disturbed auroral oval (Fig. 7c). When *B*_*tot*_ > 10 nT, σ_Φ_ occurrence is very high and the interested regions span from auroral to cap region, mainly in the daytime. A weaker occurrence stands in the evening and midnight hours within the cap (Fig. 7d).

For what concerns the scintillation on the IMF orientation, the occurrences maximizes with the larger range of *B*_*tot*_ values (>10 nT, Fig. 8d). The bulk of scintillation occurs in the polar cap, likely triggered by polar cap patches, with significant favour of the dusk sector. The intensification of the particle precipitation under negative *B*_*z*_ conditions increases the scintillation occurrence in correspondence with the auroral oval, inducing also a more pronounced cusp signature. The negative *B*_*y*_ conditions seem to be more effective in creating small scale irregularities embedded in plasma patches, whose presence maximizes the occurrence of scintillation in the dusk sector of the cap. The effect of the auroral blobs, that lead to the formation of small scale irregularities exactly at poleward boundary of the auroral oval in noon sector, is visible only when *B*_*tot*_ is large (>10 nT).

### Scintillation climatology from Galileo

**Figure 9:**
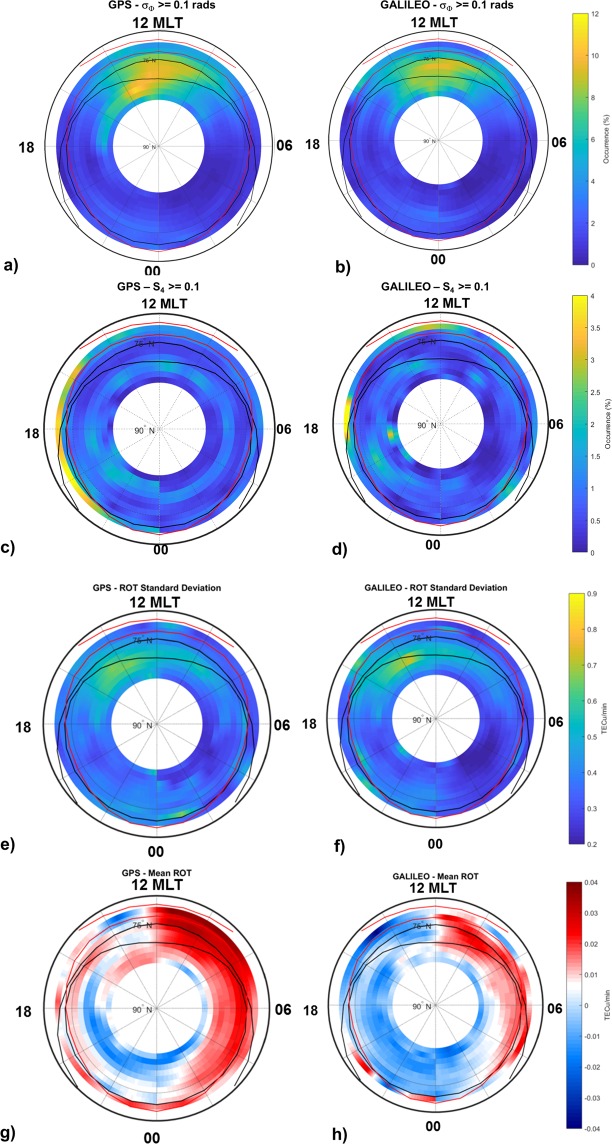
Climatology of PolaRxS data.

Figure 9 shows the ionospheric climatology along the entire 2016 derived from the Septentrio PolaRxS multi-frequency and multi-constellation (including Galileo) GNSS receiver^31^, co-located since November 2015 with the NovAtel receiver at Ny-Ålesund, Dirigibile Italia Station. Even if the Galileo constellation was not yet complete at that time (https://www.gsc-europa.eu/system-status/orbital-and-technical-parameters), its geometry of observation allows revealing additional features to the climatology of scintillations. The percentage occurrence of scintillation from weak to severe regime (σ_Φ_ ≥ 0.1 rad and S4 ≥ 0.1) exhibits differences in the post-noon sector between 14 and 18 MLT depending on the constellation considered (Galileo vs GPS), highlighting signatures in the auroral region, not visible from the climatology derived from GPS constellation (Fig. 9a).

If we compare panels e and f, we can appreciate the highest concentration of ROT standard deviation by the Galileo constellation around 14 MLT within the poleward boundary of the quiet auroral oval. In addition, the negative TEC gradients (ROT) described by the Galileo data appear to be more homogeneously distributed in the afternoon and during night-time (Fig. 9h) with respect to the map obtained using the GPS data (Fig. 9g).

## Conclusions

The climatology of ionospheric irregularities and scintillations achieved over more than 13 years of data from GPS signals sampled at 50 Hz allows drawing an unprecedented picture of the ionospheric sector observed from two GISTM GSV4004 NovAtel receivers deployed at Ny-Ålesund. To contribute to the efforts addressed to disentangle the diffractive (stochastic) from the refractive (deterministic) effects induced by the ionosphere on the GNSS signals, we have tested the use of different cut-off frequencies to look for the best choice to achieve similar behaviour of the σ_Φ_ and S4 indices occurrence over 1 year of data. This exercise demonstrated the difficulty in identifying the most suitable cut-off frequency to minimize the number of phase without amplitude scintillation events. Bearing this in mind, our climatology, based on the entire data set, was derived adopting the default cut-off frequency of 0.1 Hz to obtain a long-term assessment of the scintillations (when σ_Φ_ and S4 enhance simultaneously) and of the ionospheric irregularities producing refractive effects (when only σ_Φ_ enhances). The overall description is supported also by the analysis of the mean and of the standard deviation of ROT over 60 seconds.

Hence, we have provided a long-term scenario of the ionospheric irregularities producing or not producing L-band scintillation, giving also an assessment of the plasma dynamics over Svalbard from solar cycle 23 to 24. Our statistics showed how the magnetic pre-midnight/midnight is the most exposed to scintillations while the noon sector is the area in which the fragmentation of the plasma is less stable in space and time, giving rise to acceleration and deceleration of the irregularities.

The sorting of the climatological maps according to the different conditions of the geomagnetic field (according to Kp index) and of IMF reveals additional features. In particular:

- The refractive effects are often present in the magnetic noon, almost independently on the Kp level;
- Scintillation occurrence at pre-midnight/midnight moves from the cap toward auroral and sub-auroral latitudes when the geomagnetic perturbation enhances, following then the expansion of auroral oval during storm conditions;
- Under G3/G4/G5 storm levels, the presence of polar cap patches makes the irregularities in the polar cap effective in driving scintillation, in the pre and past midnight sector;
- A meaningful role of the polar cap patches is suggested in the scintillations occurrence happened under *B*_*z*_ negative conditions, particularly when B_*tot*_ > 5 nT.
- The prevalence of substorms in the nightside ionosphere results into an increased occurrence of scintillation in the pre- and post-midnight sectors.
- Negative *B*_*z*_ conditions results also into larger scintillation occurrence in correspondence with the auroral oval, inducing also a more pronounced cusp signature.
- Negative *B*_*y*_ conditions results in an increased probability of scintillation in the dusk sector of the cap.
- The application of the scintillation climatology to one year of data from a multi frequencies and multi constellation receiver deployed at Ny-Ålesund, confirms that the patterns in the occurrence of scintillation and irregularities not producing scintillations, and ROT features, obtained from GPS and from Galileo, generally agree.
